# Effects of ethanol on the electrochemical removal of *Bacillus subtilis* spores from water

**DOI:** 10.1186/s40201-015-0229-4

**Published:** 2015-11-18

**Authors:** Masuma Moghaddam Arjmand, Abbas Rezaee, Simin Nasseri, Said Eshraghi

**Affiliations:** Department of Environmental Health Engineering, Faculty of Medical Sciences, Tarbiat Modares University, Tehran, Iran; Department of Environmental Health Engineering, School of Public Health, and Center for Water Quality Research, Institute for Environmental Research, Tehran University of Medical Sciences, Tehran, Iran; Department of Pathobiology, School of Public Health, Tehran University of Medical Sciences, Tehran, Iran

## Abstract

This study aimed to characterize the effects of ethanol on the monopolar electrochemical process to remove *Bacillus subtilis* spores from drinking water. In particular, spores’ destruction was tested by applying 20–100 mA current for 15–60 min to *B. subtilis* spores (10^2^–10^4^ CFU/mL density), with stainless steel electrodes. The experimental results showed electrochemical removal of spores in the presence of 0.4 M ethanol at 15, 45, and 60 min and 5 mA/cm^2^ current density. However, the use of ethanol or the electrochemical process alone did not eliminate *B. subtilis* spores at these time points. Overall, this study suggests that adding ethanol to the electrochemical process successfully removes *B. subtilis* spores from drinking water.

## Introduction

*Cryptosporidium parvum* is an important microbial contaminant found in drinking water and is associated with a waterborne disease in humans [1]. Recently, *Bacillus subtilis* spores were used to evaluate the inactivation of *C. parvum* during water treatment [2]. To date, several methods of water treatment have been proposed, including chlorine. Although chlorination represents an efficient method of water treatment, it presents several disadvantages such as unfavorable taste and odor and the generation of potentially toxic disinfection products. In ddition, chlorine is ineffective when used alone against resistant microorganisms such as *C. parvum* and *Giardia* spp. [3]. A number of alternatives to chlorination have been suggested, including chemical (e.g., ozone and electrochemical treatments), physical (e.g., ultraviolet irradiation), and microwave systems [4]. In recent years, increasing attention has been paid to the electrochemical process as an alternative method to chlorination in water disinfection [5]. This treatment has been proposed since the 1950s [4] and can be divided into two categories: direct electrolyzers and mixed oxidant generators [6, 7]. The electrochemical process has several advantages, including the simplicity of the equipment and the fact that no additional chemicals are required for this method, as they can be generated during the process [8]. In the presence of iron and stainless steel electrodes, the general electrochemical mechanism for this process can be illustrated as follows:

1 $$ \mathrm{F}\mathrm{e}\ \left(\mathrm{S}\right)\to {{\mathrm{Fe}}^{+3}}_{\mathrm{aq}} + 3\ \mathrm{e}\;\left(\mathrm{Anode}\right) $$

2 $$ 3\ {\mathrm{H}}_2\mathrm{O} + 3{\mathrm{e}}^{-}\to 3/2\ {\mathrm{H}}_{2\ \mathrm{g}} + 3\ {\mathrm{OH}}^{-}\left(\mathrm{Cathode}\right) $$

Fe^3+^ and OH^−^ ions, generated at the electrodes surface, react to generate Fe(OH)_3_ compounds that can remove pollutants from aqueous solutions [9, 10]. Ethanol has also been used in water treatment processes, even though it has not been found to be an efficient sporicidal agent [11]. According to previous studies, 875 mg/L ethanol is needed to reduce *B. subtilis* populations over 6 log10 [12]. Ethanol is a membrane disrupter that induces rapid release of intracellular components and membrane disorganization, most likely due to the penetration of solvents into the hydrophobic region of the membrane bilayer. The aim of this study was to evaluate the efficiency of an electrochemical process in the presence of ethanol in water treatment. The effects of the operative parameters on *Bacillus B. subtilis* spore removal were also studied.

## Materials and methods

### Bacterial strain and culture conditions

The *B. subtilis* ATCC 6633 strain was obtained from the culture collection at the Tehran University (Iran). The strain was maintained on slant nutrient agar at 4 °C. Stocks were stored in aliquots containing 10 % glycerinated nutrient broth at −18 °C. 0.5 McFarland standards (corresponding to ~1.5 × 10^8^ CFU/mL) spores were reactivated by incubation in 100 mL Erlenmeyer flasks containing 50 mL fresh trypticase soy broth (Merck) at 37 °C for 24 h, under aerobic conditions. Next, spore suspension was poured into sterile Erlenmeyer flasks and placed in a water-bath at 80 °C for 15 min to eliminate vegetative cells. Sporulation was confirmed by optical microscopy using the Gram staining technique, and spores were diluted into water. Total counts of bacterial spore suspensions were made using the pour plate method. Briefly, after incubation at 37 °C for 48 h, spore-forming bacteria were counted, and the results were expressed as the mean number of spores/mL. Culture media and equipment were sterilized by autoclaving at 121 °C for 15 min. The pH was adjusted at 0.1 M by adding NaOH or HCl.

### Minimal inhibitory concentration (MIC)

Minimal inhibitory concentration (MIC) is defined as the lowest concentration of an antimicrobial agent that prevents the visible growth of a microorganism under certain *in vitro* condition. In this study, MICs were tested using the dilution broth method, according to the National Committee for Clinical Laboratory Standards [13]. Briefly, 1 mL of the culture was transferred into a first sterile glass tube containing 10 mL TBS medium and 0.2 to 4 M ethanol. Next, after stirring, 1 mL was transferred from the first to the second tube and so on, to obtain a total of 12 dilutions. Tubes were incubated at optimal temperature for 24 and 48 h, prior to determine the MICs.

### Experimental set up and operation

Electrochemical treatments were conducted in a single electrochemical reactor. The electrochemical reactor was equipped with two sheets of stainless steel that were used as anode and cathode electrodes. The distance between the electrodes was adjusted to 2 cm and maintained by placing plastic spacers. Experimental runs were conducted by imposing current densities ranging from 1 to 5 mA/cm^2^ (Atten APS 3005S-3, China). The electrochemical process operated in batch mode and was performed using a 500 mL capacity glass beaker with 300 mL water at room temperature. Commercially available steel plates (size 15 × 4 × 0.1 cm) were applied as electrodes and dipped in water to a depth of 10 cm. The effects of the operating conditions on the efficacy of the process were evaluated, including the applied current (50–150 mA), ethanol concentrations (0.2–0.4 M), spore concentrations (10^2^–10^4^), retention time(60 min), and current density (1–5 mA/cm^2^). The quality of water is presented in Table 1.

**Table 1:** Analysis of the water quality

Parameter	Value (mg/L)	Parameter	Value (mg/L)	Parameter	Value
Ca	59	Cl^−^	34.2	pH	7.5
Mg	20.2	SO_4_	21.4	TDS (mg/l)	364
Na	86	F^−^	0.25	Conductivity(μs)	638
K	1.25	NH_4_- N	0.1	Nitrate (mg/l)	12

The electrodes were connected to a DC power supply (Atten APS 3005S-3, China) with operational options for controlling the constant voltage and current density. The current density was calculated through the following equation as follows:

3 $$ \mathrm{C}\mathrm{D} = \mathrm{I}/\mathrm{S} $$

where I is the current through the solutions (A) and S is the area of the electrode (cm^2^). The pH level and conductivity of the solution were measured using a portable pH and EC meters (Eutech, Singapore). All the experiments were performed at a pH of 7.2. At the end of each experiment, the DC power source was switched off and the electrodes were removed from the water. During the experiments, samples were taken at 15 min interval and plated on TBS plates. All the experiments were repeated twice. The density of *B. subtilis* spore (10^2^–10^4^ m/l) removal was assessed in the electrochemical treatment using ethanol (0.2–0.4 M) at 20–100 mA current (1–5 mA/cm^2^).

## Results

**Fig. 1:**
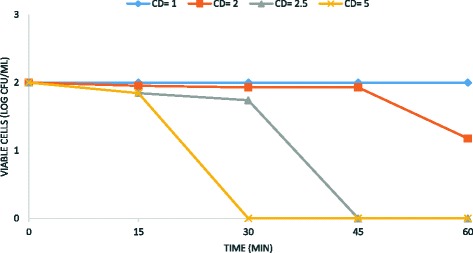
Kinetic of electrochemical *B. subtilis* spore removal from water. Experimental conditions: current density CD = 1–5 mA/cm^2^; spore density 10^2^ CFU/mL; stainless steel as electrode anode; T = 25 °C; pH = 7.2; electrodes gap = 2 cm

**Fig. 2:**
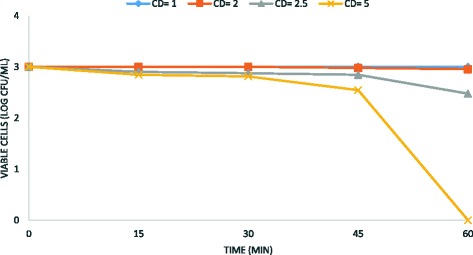
Kinetic of electrochemical *B. subtilis* spore removal from water. Experimental conditions: current density CD = 1–5 mA/cm^2^; spores density 10^3^ CFU/mL; stainless steel as electrode anode; T = 25 °C; pH = 7.2; electrodes gap = 2 cm

**Fig. 3:**
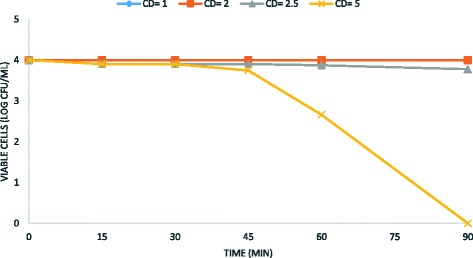
Kinetic of electrochemical *B. subtilis* spore removal from water. Experimental conditions: current density CD = 1–5 mA/cm^2^; spore density 10^4^ CFU/mL; stainless steel as electrode anode; T = 25 ° C; pH = 7.2; electrodes gap = 2 cm

In this study, the experiments were carried out in laboratory scale to evaluate the sporicidal effect of ethanol (0.2–0.4 M) in combination with the electrochemical treatment, using *B. subtilis* ATCC 6633 strain as a surrogate microorganism. In order to evaluate the sporicidal efficacy of ethanol, MIC determination was performed. The results showed that ethanol alone is not an efficient sporicidal agent (Figs. 1, 2 and 3).

**Fig. 4:**
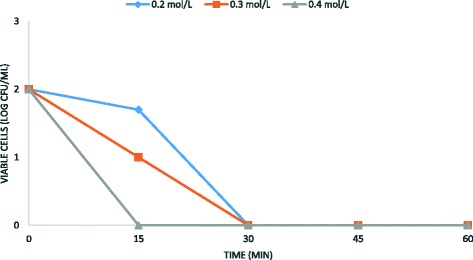
Antimicrobial efficacy of ethanol and the electrochemical treatment on *B. subtilis* spore removal from water. Experimental conditions: current density CD = 5 mA/cm^2^; spore density 10^2^ CFU/mL; ethanol concentrations 0.2–0.4 M; stainless steel as electrode anode; T = 25 °C; pH = 7.2; electrodes gap = 2 cm

**Fig. 5:**
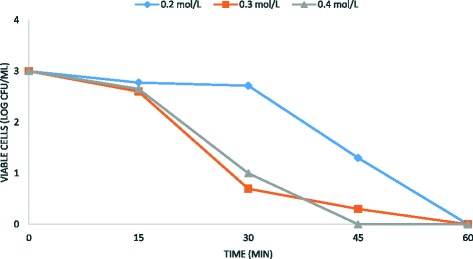
Antimicrobial efficacy of ethanol and the electrochemical treatment on *B. subtilis* spore removal from water. Experimental conditions: current density CD = 5 mA/cm^2^; spore density 10^3^ CFU/mL; ethanol concentrations 0.2–0.4 M; stainless steel as electrode anode; T = 25 °C; pH = 7.2; electrodes gap = 2 cm

**Fig. 6:**
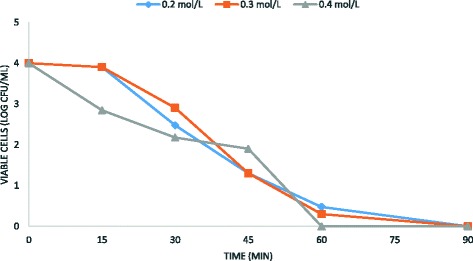
Antimicrobial efficacy of ethanol and the electrochemical treatment on *B. subtilis* spore removal from water. Experimental conditions: current density CD = 5 mA/cm^2^; spore density 10^4^ CFU/mL; ethanol concentrations 0.2–0.4 M; stainless steel as electrode anode; T = 25 °C; pH = 7.2; electrodes gap = 2 cm

However, in combination with the electrochemical treatment, it acquires a high antimicrobial activity (Figs. 4, 5 and 6).

These results demonstrated that the sporicidal efficiency is inversely proportional to the initial number of spore in solution. In addition, the sporicidal activity of the electrochemical treatment was directly proportional to ethanol concentrations, as shown in Figs. 4, 5 and 6. Overall, the results obtained in this study demonstrated that ethanol in combination with the electrochemical treatment improves the sporicidal efficiency of water disinfection, suggesting a synergistic effect between these two agents. The best result was obtained on 10^3^*B. subtilis* spores, using 0.4 M ethanol for 45 min at 5 mA/cm^2^ current density (Fig. 5).

## Discussion

Spores of *B. subtilis* are particularly resistant to conventional water disinfection treatments and, for this reason, they are used as surrogates for some waterborne pathogens such as *Cryptosporidium spp*, and as an indicator of hygienic quality of drinking water. Bacterial spores are more resistant to general sterilization and disinfection treatments such as heating, radiation, and the use of various chemicals than their vegetative cells [14]. Several parameters participate in spore resistance, including impermeability, low water content, high levels of pyridine-2,6-dicarboxylic acid and divalent cations, and outer membrane thickness [15]. In addition, spore DNA is protected against various types of damage [16]. In recent years, increasing attention has been paid to electrochemical oxidation as an efficient technology for water disinfection. During this process, free chlorine is produced. This chemical damages the bacterial outer membrane, penetrates into the periplasm, destroys the inner membrane and degenerates cytoplasmic proteins. Also, the process can oxidize the microbes on the electrode surfaces [17]. Usually, the oxidants of the electrochemical treatment are reactive oxygen species generated from the oxidation of water molecules [18]. It was shown that the electrochemical treatment efficiently removes bacteria spores but not their vegetative cells [19]. The aim of this study was to evaluate the efficiency of a combination of the electrochemical process and ethanol at low concentrations for disinfection of *B. subtilis* spores. Ethanol is a general bactericidal agent and has been widely applied for disinfection of human tissues and contaminated surfaces. According to the presented results, however, ethanol alone does not possess sporicidal activity [11]. It has been reported that ethanol causes microbial membrane damage and denaturation of proteins thus interfering with cell metabolism and inducing cell lysis. According to the obtained results, maximum sporicidal effects were obtained by adding 0.4 M ethanol to10^4^*B. subtilis* spores at 100 mA current. Lower ethanol concentration (0.2 M) increased the reaction time to 90 min. Ethanol alone does not possess high sporicidal efficiency. Some studies have reported that the combination of ethanol with ferric chloride and ethylenediaminetetraacetic acid can act as sporicidal agent [20]. Also, it has been shown that ethanol and anionic surfactants have sporicidal activity at low pH values. In this study, the sporicidal effects on *B. subtilis* spores was assessed by MIC. The results showed that ethanol alone has not sporicidal effect on *B. subtilis* spores. The electrochemical treatment exerted a low sporicidal effect on small numbers of *B. subtilis* spores, but it failed for large number of spores. These results indicated that the electrochemical treatment is an efficient method for water disinfection, but is not sufficient to remove disinfectant-resistant bacteria such as spore forming bacteria. This study showed that ethanol significantly increases the sporicidal efficiency of the electrochemical process.

## Conclusion

The results obtained in this study show that *B. subtilis* spores were killed at 90 min by electrochemical water disinfection using ethanol. It was observed that increasing the operational time and adding ethanol to the electrochemical process improved the spore removal efficiency. Moreover, increasing the supporting electrolyte concentration in the solution reduces the specific electrical energy consumption.
